# Thickness of Extraocular Muscle and Orbital Fat in MRI Predicts Response to Glucocorticoid Therapy in Graves' Ophthalmopathy

**DOI:** 10.1155/2017/3196059

**Published:** 2017-08-06

**Authors:** Lingling Xu, Linna Li, Cuihua Xie, Meiping Guan, Yaoming Xue

**Affiliations:** Department of Endocrinology, Nanfang Hospital, Southern Medical University, Guangzhou, Guangdong 510515, China

## Abstract

33 patients with active, moderate-severe Graves' ophthalmopathy (GO) received 4.5 g methylprednisolone for 12 weeks and were divided by efficacy into two groups (responsive and unresponsive). All patients and 10 controls underwent orbital MRI examination at baseline. No significant difference was seen in baseline clinical characteristics between the two GO groups. The values of exophthalmos were higher in both GO groups than in the control and were higher in the responsive group versus the unresponsive group. Compared to the unresponsive group, the responsive group had a thicker inferior rectus as well as thinner orbital fat. The inferior rectus/fat ratio was significantly higher in the responsive group versus the unresponsive group. Multivariate logistic regression analysis showed that the exophthalmos value and inferior rectus/fat ratio were significantly associated with the response to glucocorticoid (GC). ROC analysis revealed that the cut-off points of the inferior rectus/fat ratio combined with the exophthalmos value to indicate efficacy were 1.42 and 20.78. For moderate-severe GO patients with CAS > 3, the combined inferior rectus/fat ratio and exophthalmos value in MRI may be a valuable indicator to predict the response to GC therapy.

## 1. Introduction

Graves' ophthalmopathy (GO) is an autoimmune disease of the orbit occurring in 25–50% patients with Graves' disease (GD) [1], which significantly reduces the patients' quality of life. The classic ocular signs of GO include proptosis, eyelid retraction, periorbital edema, diplopia, and visual loss.

The natural course of GO consists of two stages: an active inflammatory stage and a static stage. The anti-inflammatory treatment is primarily used in the first stage [1], and intravenous glucocorticoid (GC) is currently recommended as the first-line therapy for active and moderate-severe cases [2–4]. However, the treatment response is shown to vary considerably between individuals; even though the patients underwent rigorous screening and standard treatment, the overall response rate was only 65–80% [4–6].

An accurate evaluation of GO activity is important for predicting a response to anti-inflammatory treatment [7]. Mourits et al. [8] established a clinical activity score (CAS) which is correlated with the treatment response, and if CAS > 3, immunosuppressive therapy is recommended [2]. However, in a clinical setting, the CAS assessment is not clearly elucidated in many GO patients, rendering difficulty in making adequate therapeutic decisions. Some studies have shown that imaging of orbital tissues in GO provides valuable information for both diagnosis and treatment decisions [9–12].

Therefore, objective modalities such as orbital MR imaging (MRI) should be applied appropriately to improve the accuracy of diagnosis [13]. As GO is characterized by the swelling of extraocular muscles and orbital fat [14], we hypothesized that the thickness of extraocular muscles and orbital fat detected by MRI might be useful in evaluating treatment response.

In the present study, the moderate-severe GO patients with CAS > 3 were treated with GC and were divided into two groups according to the therapeutic effect. We attempted to investigate the differences in baseline parameters of the orbital MRI between the two groups, which may provide a deeper insight while predicting response to GC treatment.

## 2. Methods

### 2.1. Subjects

A total of 42 active and moderate-severe GO patients in Nanfang Hospital, Southern Medical University, were enrolled between January 2016 and July 2016, and 33 subjects were eligible after screening for inclusion and exclusion criteria. In addition, 10 normal volunteers (4 males and 6 females) were recruited as controls, who should meet the following criteria: no history of thyroid disease; normal thyroid function and thyroid-related antibodies in blood test; and no symptoms or signs of exophthalmos, diplopia, swelling, pain, itches, photophobia, lacrimation, foreign body sensation, conjunctival congestion, and decreased eye mobility.

The diagnosis of active and moderate-severe GO was based on the European Group on Graves' Orbitopathy (EUGOGO) consensus [2, 4]. CAS ≥ 3 was defined as active GO, and disease severity was also assessed by the severity scales of EUGOGO [15].

Inclusion criteria are as follows: (1) patients were aged between 18 and 65; (2) GO duration was shorter than 18 months; (3) thyroid function was under control (defined by normal FT4 and TSH < 4.78).

Exclusion criteria are as follows: (1) monocular involvement; (2) vision-threatening GO; (3) patients with optic atrophy, cataracts, or retinopathy; (4) received intravenous or oral GC within 6 months; (5) received retroocular radiotherapy within 6 months; (6) glaucoma; (7) pregnancy or lactating women; (8) abnormal liver function (transaminases exceeding 2-fold of the upper limit of the normal range) or abnormal renal function (serum creatinine > 136 *μ*mol/L); (9) history of peptic ulcer; (10) history of heart failure, coronary heart disease, and stroke; (11) poor control of diabetes, HbA1c > 8%; (12) osteoporosis; (13) active infection (especially tuberculosis); (14) malignant tumor; (15) unable to complete the entire course of treatment.

All the GO patients received 4.5 g methylprednisolone for 12 weeks as recommended [4, 16]. The protocol was as follows: 0.5 g methylprednisolone weekly for 6 weeks, followed by 0.25 g methylprednisolone weekly for 6 weeks. All participants signed an informed consent. The study protocol was approved by the Ethics Committee of Nanfang Hospital, Southern Medical University. The study was registered at the Chinese Clinical Trial Registry (ChiCTR-RPC-16008209).

### 2.2. Grouping

CAS and severity assessment were determined by the same endocrinologist who had been trained in ophthalmology before and 6 months after the end of treatment. The patients were divided into two groups “the responsive group” and “the unresponsive group” based on efficacy. “The responsive group” was defined as CAS decreased by a minimum of 2 points and CAS < 3/7, together with at least one of the following parameters improved, without worsening of the other parameters: (1) reduction of proptosis minimally by 2 mm; (2) reduction of lid width by at least 2 mm; (3) decrease in the Gorman score (from constant to inconstant, inconstant to intermittent, and intermittent to absent); (4) improvement of visual acuity by at least one Snellen line. “The unresponsive group” was defined as CAS dropping to less than 2 points or staying active (CAS ≥ 3/7).

### 2.3. Orbital MRI

All GO patients and controls underwent orbital MRI examination at baseline. The MRI scans were performed using a Siemens Magnetism Vision Plus 1.5T MR scanner with the patients in a supine position. The orbit was scanned using transverse, coronal and oblique sagittal position that was parallel to the optic nerve.

Images were acquired with the following parameters: spin echo (SE) sequence T2WI: TR 5000 ms, TE 128 ms; SE sequence T1WI: TR 390 ms, TE 6 ms; SE double back wave sequence: TR 4020 ms, and TE 22/99 ms; the fat suppression sequence was also performed. Section thickness are as follows: 3.0 mm, intersection gap 0.3 mm, and time of acquiring data: 1 time.

**Figure 1:**
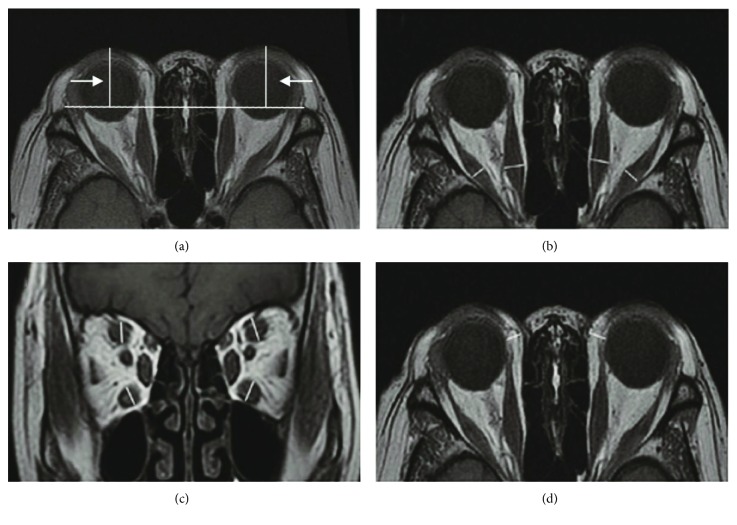
Methods for ophthalmic parameter measurement. (a) The values of exophthalmos. (b) The horizontal diameters of the medial and lateral rectus muscles. (c) The vertical diameters of the superior and inferior rectus muscles. (d) The thickness of fatty tissue.

The following are the methods for ophthalmic parameter estimation (Figure 1):

- The value of exophthalmos: in the T1WI cross section, we selected the maximum display level of the eyeball and optic nerve. The vertical distance measured between the anterior border of the corneal and bilateral lines of zygomatic arch is the value of exophthalmos (Figure 1(a)).
- The thickness of extraocular muscles: we evaluated the thickness (mm) of each extraocular muscle (superior, inferior, lateral, and medial rectus). The horizontal diameters of the medial and lateral rectus and the vertical diameters of the superior and inferior rectus were measured on the series of images; the largest diameter of the middle section of each muscle was selected for further comparison. The sum thickness of the four (superior, inferior, lateral, and medial rectus) extraocular muscles was also calculated (Figures 1(b) and 1(c)).
- The thickness of fatty tissue: the largest diameter of the middle section of the medial and lateral rectus and optic nerve in transverse T1WI was chosen. The thickness of the fatty tissue was defined as the maximum thickness from the medial wall of the eyeball (or the lateral margin of the medial rectus) to the medial wall of the orbit [17, 18] (Figure 1(d)).

### 2.4. Statistical Analysis

The IBM SPSS statistics (V.19.0, IBM Corp., USA, 2010) was used for data analyses. For each patient, the mean value of the two eyes was presented for each ophthalmological parameter. Results were expressed as mean ± SD for normally distributed data and median with interquartile range for nonnormally distributed data or n (%). The normally distributed values were analyzed by Student's *t*-test for differences between the two groups. One-way ANOVA was used to analyze the differences among three groups. The differences between the groups were analyzed by the Kruskal-Wallis test for nonparametric values. Pearson's *χ*^2^ test was employed to analyze the categorical data. Multivariate logistic regression was performed to determine the correlation between the clinical parameters and the response to GC. ROC curve analysis was conducted to determine the sensitivity and specificity of the inferior rectus thickness/orbital fat thickness ratio combined with exophthalmos value for predicting the curative effect. All *P* values reported were two tailed, and *P* value < 0.05 was considered statistically significant while <0.001 was highly significant.

## 3. Results

### 3.1. Baseline Characteristics of the Patients

A total of 33 GO patients and 10 normal controls were enrolled for participation in the trial.

Table 1 summarizes the baseline clinical characteristics of each group, and no significant differences between the two GO treatment groups were observed (all *P* > 0.05).

### 3.2. Comparison of Ophthalmic Parameter Measurement in MRI

All measurements were done by the same observer.

#### 3.2.1. Values of Exophthalmos (Table 2, Figure 2(a))

The values of exophthalmos among the three groups differed significantly (*P* < 0.001). The values of exophthalmos were higher in both GO groups as compared to those in the control (*P* < 0.001), and the values of exophthalmos were higher in the responsive group than in the unresponsive group (*P* < 0.05).

#### 3.2.2. Thickness of Extraocular Muscles (Table 2, Figure 2(b))

Comparing the treated GO patients to the control, we found that the thickness of each extraocular muscle was significantly higher in both GO groups than in the control (*P* < 0.05 and <0.001). However, only the thickness of the inferior rectus was higher in the responsive group than in the unresponsive group (*P* < 0.05). No statistically significant differences were seen in the thickness of the other extraocular muscles between the two GO groups.

#### 3.2.3. Sum Thickness of Extraocular Rectus Muscles (EOM) (Table 2, Figure 2(c))

We also calculated the sum thickness of the inferior, medial, superior, and lateral rectus. The thickness of EOM was significantly increased in both GO groups as compared to that in the control (both *P* < 0.01), and the thickness of EOM was significantly increased in the responsive group than in the unresponsive group (*P* < 0.05).

#### 3.2.4. Thickness of Fatty Tissue (Table 2, Figure 2(d))

The thickness of fatty tissue was higher in both the responsive (*P* < 0.05) and unresponsive (*P* < 0.001) groups compared to that in the control. However, the orbital fat thickness was significantly decreased in the responsive group than in the unresponsive group (*P* < 0.05).

#### 3.2.5. Inferior Rectus Thickness/Orbital Fat Thickness (Inferior Rectus/Fat Ratio) (Table 2, Figure 2(e))

Based on the above findings, only the thickness of the inferior rectus was higher in the responsive group than in the unresponsive group (*P* < 0.001). Thus, we speculated that the inferior rectus/fat ratio might be a suitable indicator for predicting response. Although there was no difference in the ratio between the unresponsive and control groups (*P* > 0.05), the ratio was significantly higher in the responsive group as compared to that in the unresponsive group (*P* < 0.001).

#### 3.2.6. EOM Thickness/Orbital Fat Thickness (EOM/Fat Ratio) (Table 2, Figure 2(e))

We also calculated the EOM/fat ratio and found that there was no significant difference between the unresponsive group and control (*P* > 0.05). Also, the ratio was significantly higher in the responsive group than in the unresponsive group (*P* < 0.001).

### 3.3. Predictive Factors for Response to GC Treatment in GO Patients

Due to high intercorrelation between the inferior rectus/fat ratio and the EOM/fat ratio (*P* < 0.01, data not shown), the EOM/fat ratio was excluded from multivariate logistic regression in order to avoid multicollinearity.

We performed multivariate logistic regression analysis with the response to GC as a dependent variable, and age, gender, smoking history, BMI, SBP, DBP, duration of GO, antithyroid treatments, FT3, FT4, TSH, TRAb, CAS, exophthalmos value, and inferior rectus/fat ratio as independent variables.

Exophthalmos value (OR 0.33, 95% CI: 0.12–0.88, *P* = 0.027) and inferior rectus/fat ratio (OR 0.001, 95% CI: 0.001–0.11, *P* = 0.016) were found to be significantly associated with the response to GC in GO patients (Table 3).

### 3.4. ROC Curve Analysis

ROC analysis was performed to identify the optimal cut-off point of the inferior rectus/fat ratio and exophthalmos value for predicting the response to GC in GO patients. The result revealed that the area under the curve (AUC) analysis of the exophthalmos value was not statistically significant (AUC = 0.65; 95% CI: 0.45–0.84; *P* = 0.15), which indicated that the exophthalmos value could not be a sole independent factor. Therefore, we performed the ROC analysis to investigate the optimal cut-off points of the inferior rectus/fat ratio combined with the exophthalmos value to indicate the treatment response.

Furthermore, we repeated the multivariate logistic regression analysis with the response to GC as a dependent variable, whereas the inferior rectus/fat ratio and exophthalmos value were as independent variables. Using this approach, we obtained the predicted probability of a parameter of the combined inferior rectus/fat ratio and exophthalmos value, which was used for multivariable ROC analysis as an independent value.

**Figure 3:**
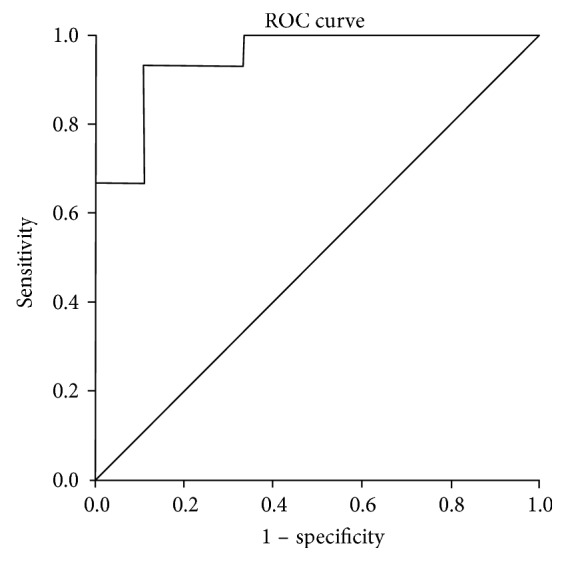
ROC analysis of the inferior rectus/fat ratio combined with the exophthalmos value to indicate response to GC in GO patients. (AUC = 0.95; 95% CI: 0.88–1.00; sensitivity, 86.7%; specificity, 88.9%, *P* < 0.001). Cut-off point of the inferior rectus/fat ratio: 1.42; cut-off point of the exophthalmos value: 20.78.

The cut-off points of the inferior rectus/fat ratio combined with the exophthalmos value were revealed as the response to GC: 1.42 and 20.78, respectively (AUC = 0.95; 95% CI: 0.88–1.00; sensitivity, 86.7%; specificity, 88.9%, *P* < 0.001) (Figure 3).

## 4. Discussion

While treating GO patients, the severity and activity of the disease should be assessed. The severity assessment can aid in determining the worth of risk in active treatment, and activity evaluation can be valuable in the selection of immunosuppressive therapy or surgical treatment, as the former is recommended in the active inflammatory stage [2, 4].

Among the several means of assessing GO activity proposed over the past few decades, CAS is a simple and widely used method [2, 8], which can describe congestion and edema of the eyelids, conjunctiva, and lacrimal caruncle and by questioning the patient about stationary or moving eye pain can help to judge the presence of acute inflammatory state of the orbit. However, the disadvantage of CAS is its subjectivity; thus, it can only be qualitative and cannot directly show internal orbital lesions.

EUGOGO in 2008 and 2016 unanimously recommended that for patients with moderate-severe and active exophthalmos, intravenous GC treatment can be used as first-line therapy [2, 4]. In the present study, we used the recommended regimen: a total dose of 4.5 g GC therapy lasting for 12 weeks [4–6].

However, in a clinical setting, we find that quite a few patients with moderate-severe GO and CAS > 3 do not achieve a significant improvement after standard treatment. Several years ago, EUGOGO carried out a large, multicenter RCT study and compared the efficacy and side effects of 3 different cumulative doses of methylprednisolone [5]. This study demonstrated that the moderate doses of GC (5 g) treatment of GO were an effective method; however, some patients continued to be unresponsive to the treatment. Furthermore, according to previous studies, many patients were dissatisfied with the outcomes of the treatment [15, 19, 20].

The imperfectness of the existing treatment methods may be attributed to the following reasons: (1) the pathogenesis of GO has not been fully elucidated, and thus, it cannot receive targeted therapy; (2) conducting a large-scale RCT study to compare different treatment options is difficult due to the low incidence and prevalence of GO; (3) nonprofessional evaluation of the activity and severity may lead to a situation wherein active GO does not receive timely treatment, and inactive GO has treated extremely aggressively with poor response [19]. Therefore, an optimal method to judge the activity of GO and predict the response of GC accurately and effectively is a prerequisite. A recent study [21] aimed to find a reliable and easily accessible biomarker to predict the outcome of GC, and the result showed that a parameter of the combined serum miR-224-5p and TRAb could effectively predict GC sensitivity in GO patients. However, miR-224-5p is not a routine test in clinical practice.

In the present study, the moderate-severe GO patients with CAS > 3 were treated with GC, and the patients were grouped according to the treatment response. The responsive and unresponsive groups showed similar baseline parameters, including age, gender, smoking rate, disease duration, thyroid function, TRAb levels, and CAS score. We speculated that some objective examinations, such as orbital MRI before the initiation of treatment, might play a major role in the assessment of disease activity and treatment response. Orbital MRI can clearly show the anatomical structure of the extraocular muscles, orbital fat, and optic nerve and can be used for the quantitative analysis of some parameters, such as the degree of exophthalmos, thickness or volume of extraocular muscle, and orbital fat, as well as signal values. Furthermore, we attempted to identify the baseline differences in orbital MRI parameters between the two treated GO groups, which may support the prediction of treatment response.

To date, little attention has been given to the differential involvement of extraocular muscle and orbital fat in GO patients. A previous study [12] found that an increase in fat volume (FV) was characterized by proptosis while muscle enlargement was associated with older age, higher TBII values, more proptosis, and impaired motility. Another study [11] proposed that increased FV can be associated with a rise in proptosis and longer GO duration while increased muscle volume (MV) was associated with older age, severe GO, high TBII, and current smoking. Furthermore, Naik et al. [22] showed three predominant forms of soft tissue involvement in GO, that include predominantly fat expansion, predominantly muscle enlargement, and a combination of both and suggested that the second form was more sensitive to GC. These results suggest that enlarged muscles might be correlated with the increased disease activity and severity.

In the present study, we only measured the thickness but not the volume of the extraocular muscles and orbital fat. However, most of the previous studies on the orbital tissue of GO measured the volume [11, 23], which requires the use of Mimics, which is an image processing software with three-dimensional visualization. This method is rather complicated and challenging for clinicians to evaluate the orbital tissue promptly. Notably, as fat is filled with every corner of the orbit lacking specific forms and as the orbital volume is fixed, it can increase and migrate through the orbital space surrounding the eyeball. Thus, according to previous studies [17, 18], we deduced that the maximum fat thickness on the inner side of the eyeball may indicate changes of the orbital FV, which is a relatively straightforward and reliable method.

A previous study showed that the most obvious pathological change within the orbit in GO patients is the enlargement of extraocular muscles [9] and inferior rectus is the most often involved [10, 24, 25]. Our study found that in comparison to the unresponsive group, the responsive group had a significantly thicker inferior rectus while no significant difference in the thickness was found in the other extraocular muscles. Furthermore, we also found that the orbital fat thickness in the responsive group was significantly decreased than that in the unresponsive group. Therefore, compared to the unresponsive group, the responsive group had a thicker inferior rectus as well as thinner orbital fat. As aldosterone-renin ratio is recommended as the most reliable tool available for screening primary aldosteronism, we speculated that for moderate-severe GO patients with CAS > 3, inferior rectus/fat ratio may be a useful indicator to predict the response of GC therapy. ROC analysis showed that the parameter of the combined inferior rectus/fat ratio and exophthalmos value could effectively predict the GC response in GO patients. In case the inferior rectus/fat > 1.42 combined with exophthalmos value > 20.78 existed, the patients may exhibit a superior response to GC treatment, thereby providing a simple and feasible evaluation method for clinicians.

## Figures and Tables

**Figure 2:**
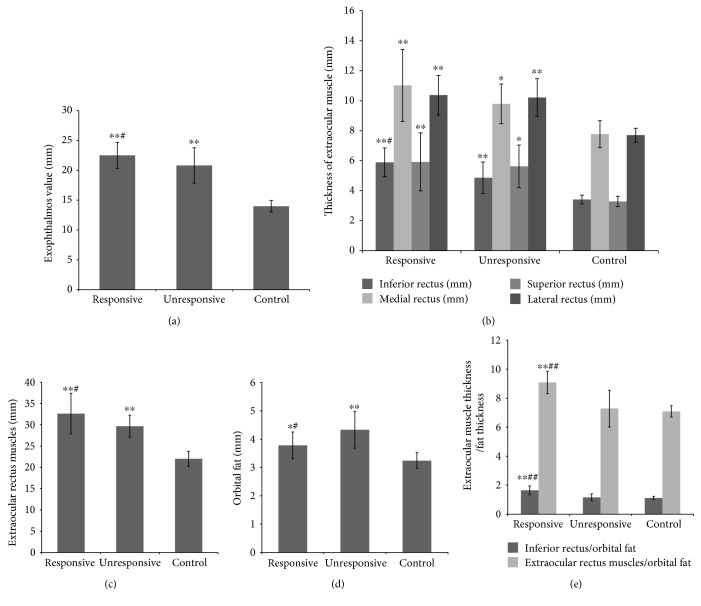
Comparison of ophthalmic parameter measurement in MRI. ^∗^*P* < 0.05 versus the control group; ^∗∗^*P* < 0.001 versus the control group; ^#^*P* < 0.05 versus the unresponsive group; ^##^*P* < 0.001 versus the unresponsive group.

**Table 1:** Clinical features of each group.

	Responsive (*n* = 18)	Unresponsive (*n* = 15)	Control (*n* = 10)	*P* value
Age (years)	42.83 ± 12.28	44.33 ± 10.79	45.70 ± 13.66	0.83
Female (*n*, %)	11 (61.1%)	8 (53.3%)	6 (60.0%)	0.95
Smoking history	4 (22.2%)	3 (20%)	2 (20%)	0.99
Alcohol intake	2 (11.1%)	1 (6.7%)	1 (10%)	0.91
BMI	22.75 ± 2.11	24.57 ± 3.88	22.01 ± 1.27	0.06
SBP (mmHg)	133.11 ± 17.10	131.40 ± 17.15	132.30 ± 15.79	0.96
DBP (mmHg)	77.56 ± 10.22	83.40 ± 9.46	80.70 ± 10.76	0.26
ALT (U/L)	15.67 ± 7.74	20.56 ± 14.41	18.20 ± 5.16	0.40
AST (U/L)	20.39 ± 7.95	21.25 ± 8.03	21.90 ± 6.15	0.88
BUN (mmol/L)	4.70 ± 1.49	3.95 ± 0.96	4.53 ± 1.12	0.22
Cr (*μ*mol/L)	55.89 ± 22.78	44.27 ± 15.46	56.30 ± 5.89	0.13
TG (mmol/L)	1.39 ± 0.70	1.50 ± 0.74	1.75 ± 0.69	0.48
TC (mmol/L)	4.85 ± 1.19	5.15 ± 1.49	4.69 ± 1.33	0.72
HDL (mmol/L)	1.24 ± 0.47	1.20 ± 0.37	1.10 ± 0.28	0.70
LDL (mmol/L)	2.86 ± 0.69	3.23 ± 1.11	2.81 ± 1.00	0.52
FT3 (pg/mL)	5.75 ± 6.31	4.23 ± 1.77	2.99 ± 0.39	0.25
FT4 (ng/dL)	1.62 ± 1.17	1.43 ± 0.54	1.15 ± 0.16	0.36
TSH (mUI/L)	0.468 (0.004–2.465)	0.070 (0.004–1.15)	2.330 (1.304–3.05)	0.02^∗^
TRAb (UI/L)	12.75 (5.78–27.20)	12.75 (5.59–22.09)	0.580 (0.320–0.650)	<0.001^∗∗^
Hyperthyroidism duration (months)	14 (7.25–25.5)	22 (12–42)	—	0.49
GO duration (months)	8.0 (3–12.25)	9.5 (4.0–12.5)	—	0.66
*Antithyroid treatments*				
Antithyroid drug	6 (33.3%)	7 (46.7%)	—	0.44
Radioiodine	9 (50.0%)	6 (40.0%)	—	0.57
Thyroidectomy	3 (16.7%)	2 (13.3%)	—	0.79
CAS	4.56 ± 1.10	4.27 ± 1.03	—	0.45
Lid width (mm)	11.06 ± 1.12	10.67 ± 1.02	8.50 ± 0.86	<0.001^∗∗^
Visual acuity	0.80 ± 0.15	0.83 ± 0.18	0.99 ± 0.17	0.02^∗^
*Diplopia*				0.02^∗^
Absent	6 (33.3%)	4 (26.7%)	10 (100%)	
Intermittent	5 (27.8%)	5 (33.3%)	0	
Inconstant	4 (22.2%)	4 (26.7%)	0	
Constant	3 (16.7%)	2 (13.3%)	0	

^∗^ *P* < 0.05; ^∗∗^*P* < 0.001. TSH: responsive group versus unresponsive group, *P* = 0.722. TRAb: responsive group versus unresponsive group, *P* = 0.874. Lid width: responsive group versus unresponsive group, *P* = 0.288. Visual acuity: responsive group versus unresponsive group, *P* = 0.558. Diplopia: responsive group versus unresponsive group, *P* = 0.954.

**Table 2:** Comparison of ophthalmic parameter measurement in MRI.

	Responsive (*n* = 18)	Unresponsive (*n* = 15)	Control	*P*
Exophthalmos value (mm)	22.50 ± 2.17^∗∗^^#^	20.82 ± 2.96^∗∗^	13.98 ± 0.97	<0.001
Inferior rectus (mm)	5.89 ± 0.96^∗∗^^#^	4.86 ± 1.05^∗∗^	3.41 ± 0.29	<0.001
Medial rectus (mm)	11.02 ± 2.41^∗∗^	9.79 ± 1.32^∗^	7.77 ± 0.90	<0.001
Superior rectus (mm)	5.92 ± 1.93^∗∗^	5.62 ± 1.42^∗^	3.28 ± 0.35	<0.001
Lateral rectus (mm)	10.37 ± 1.32^∗∗^	10.21 ± 1.26^∗∗^	7.70 ± 0.46	<0.001
EOM (mm)	32.62 ± 4.78^∗∗^^#^	29.67 ± 2.59^∗∗^	22.02 ± 1.76	<0.001
Orbital fat (mm)	3.78 ± 0.47^∗^^#^	4.33 ± 0.65^∗∗^	3.24 ± 0.28	<0.001
Inferior rectus/fat ratio	1.65 ± 0.30^∗∗^^##^	1.16 ± 0.24	1.12 ± 0.11	<0.001
EOM/fat ratio	9.09 ± 0.77^∗∗^^##^	7.29 ± 1.27	7.09 ± 0.39	<0.001

^∗^ *P* < 0.05 versus control group; ^∗∗^*P* < 0.001 versus control group; ^#^*P* < 0.05 versus unresponsive group; ^##^*P* < 0.001 versus unresponsive group.

**Table 3:** Predictive factors for response to GC in GO patients.

Variables	Odd ratio (95% CI)	*P* value
Exophthalmos value	0.33 (0.12–0.88)	0.027
Inferior rectus/fat ratio	0.001 (0.001–0.11)	0.016
